# Supplementary material for: Non-Equilibrium Thermodynamics View on Kinetics of Autocatalytic Reactions—Two Illustrative Examples
Source: Molecules. 2021 Jan 22;26(3):585. doi: 10.3390/molecules26030585 (PMC7866015; doi:10.3390/molecules26030585)
Supplement: Supplementary file 1 [file molecules-26-00585-s001.pdf]

Supplementary material

Non-equilibrium thermodynamics view on kinetics of autocatalytic reactions – two illustrative examples

Miloslav Pekař

Faculty of Chemistry, Brno University of Technology, Purkyňova 118, 612 00 Brno, Czech Republic

E-mail: pekar@fch.vut.cz

The used thermodynamic method for the derivation of the thermodynamic polynomial (rate equation) originated in a paper by Samohýl and Malijevský [16] and was further developed in subsequent work, particularly in [9, 10].

Single reaction.

The third-degree polynomial approximating the rate function [9] (p. 251), [10] is in our case (component numbers instead of symbols are used):

$J = k_{000} + k_{100}c_1 + k_{010}c_2 + k_{001}c_3 + k_{200}\,c_1^2 + k_{020}\,c_2^2 + k_{002}\,c_3^2 + k_{110}c_1c_2 + k_{101}c_1c_3 + k_{011}c_2c_3 + k_{300}\,c_1^3 + k_{030}\,c_2^3 + k_{003}\,c_3^3 + k_{111}c_1c_2c_3 + k_{210}c_1^2c_2 + k_{201}c_1^2c_3 + k_{021}c_2^2c_3 + k_{120}c_1c_2^2 + k_{102}c_1c_3^2 + k_{012}c_2c_3^2.$ (A1)

The polynomial coefficients k_{ijl} are functions of temperature only and in the end they represent rate coefficients or constants [9] (p. 251), [10]. In equilibrium, where $J = 0$, substituting for $c_{2,\text{eq}}$ from (4), we have:

$0 = k_{000} + k_{100}c_{1,\text{eq}} + k_{200}\,c_{1,\text{eq}}^2 + k_{300}\,c_{1,\text{eq}}^3 + \left[k_{001} + (k_{010}K^{-1} + k_{101})c_{1,\text{eq}} + (k_{110}K^{-1} + k_{201})c_{1,\text{eq}}^2 + (k_{210}K^{-1} + k_{120}K^{-2})c_{1,\text{eq}}^3\right]c_{3.\text{eq}} + \left[k_{002} + (k_{020}\,K^{-2} + k_{111}K^{-1})c_{1,\text{eq}}^2 + (k_{011}K^{-1} + k_{102})c_{1,\text{eq}}\right]c_{3,\text{eq}}^2 + \left[k_{003} + k_{030}\,K^{-3}c_{1,\text{eq}}^3 + k_{012}K^{-1}c_{1,\text{eq}} + k_{021}K^{-2}c_{1,\text{eq}}^2\right]c_{3,\text{eq}}^3.$ (A2)

Identity (A2) should be valid in any equilibrium, consequently, expressions in round brackets and following coefficients are zero [9] (p. 251), [10]:

$k_{000} = k_{100} = k_{200} = k_{300} = k_{001} = k_{002} = k_{003} = k_{030} = k_{012} = k_{021} = 0.$ (A3)

From the zero expressions we can derive:

$k_{101} = -k_{010}K^{-1},\ \ k_{201} = -k_{110}K^{-1},\ \ k_{210} = -k_{120}K^{-1},\ \ k_{111} = -k_{020}\,K^{-1},\ \ k_{102} = -k_{011}K^{-1}.$ (A4)

Substituting from (A3) and (A4) into (A1), thermodynamic polynomial (5) is obtained.

Lotka's scheme.

The full approximating third-degree polynomial contains 120 terms; the numbering of the chemical components is the same as in the main text: $\text{AX} = 1, \text{X} = 2, \text{A} = 3, \text{Y} = 4, \text{BY} = 5, \text{BX} = 6, \text{Z} = 7$. The polynomial is as follows (superscripts at concentrations mean powers):

$\mathbf{J} = \mathbf{k}_{0000000} + \mathbf{k}_{1000000}c_1 + \mathbf{k}_{0100000}c_2 + \mathbf{k}_{0010000}c_3 + \mathbf{k}_{0001000}c_4 + \mathbf{k}_{0000100}c_5 + \mathbf{k}_{0000010}c_6 + \mathbf{k}_{0000001}c_7 + \mathbf{k}_{2000000}c_1^2 + \mathbf{k}_{0200000}c_2^2 + \mathbf{k}_{0020000}c_3^2 + \mathbf{k}_{0002000}c_4^2 + \mathbf{k}_{0000200}c_5^2 + \mathbf{k}_{0000020}c_6^2 + \mathbf{k}_{0000001}c_7^2 + \mathbf{k}_{1100000}c_1c_2 + \mathbf{k}_{1010000}c_1c_3 + \mathbf{k}_{1001000}c_1c_4 + \mathbf{k}_{1000100}c_1c_5 + \mathbf{k}_{1000010}c_1c_6 + \mathbf{k}_{1000001}c_1c_7 + \mathbf{k}_{0110000}c_2c_3 + \mathbf{k}_{0101000}c_2c_4 + \mathbf{k}_{0100100}c_2c_5 + \mathbf{k}_{0100010}c_2c_6 + \mathbf{k}_{0100001}c_2c_7 +$

$$
\begin{aligned}
&\mathbf{k}_{0011000}c_3c_4 + \mathbf{k}_{0010100}c_3c_5 + \mathbf{k}_{0010010}c_3c_6 + \mathbf{k}_{0010001}c_3c_7 + \mathbf{k}_{0001100}c_4c_5 + \mathbf{k}_{0001010}c_4c_6 + \\
&\mathbf{k}_{0001001}c_4c_7 + \mathbf{k}_{0000110}c_5c_6 + \mathbf{k}_{0000101}c_5c_7 + \mathbf{k}_{0000011}c_6c_7 + \mathbf{k}_{3000000}c_1^3 + \mathbf{k}_{0300000}c_2^3 + \\
&\mathbf{k}_{0030000}c_3^3 + \mathbf{k}_{0003000}c_4^3 + \mathbf{k}_{0000300}c_5^3 + \mathbf{k}_{0000030}c_6^3 + \mathbf{k}_{0000003}c_7^3 + \mathbf{k}_{2100000}c_1^2c_2 + \\
&\mathbf{k}_{2010000}c_1^2c_3 + \mathbf{k}_{2001000}c_1^2c_4 + \mathbf{k}_{2000100}c_1^2c_5 + \mathbf{k}_{2000010}c_1^2c_6 + \mathbf{k}_{2000001}c_1^2c_7 + \mathbf{k}_{1200000}c_1c_2^2 + \\
&\mathbf{k}_{0210000}c_2^2c_3 + \mathbf{k}_{0201000}c_2^2c_4 + \mathbf{k}_{0200100}c_2^2c_5 + \mathbf{k}_{0200010}c_2^2c_6 + \mathbf{k}_{0200001}c_2^2c_7 + \mathbf{k}_{1020000}c_1c_3^2 + \\
&\mathbf{k}_{0120000}c_2c_3^2 + \mathbf{k}_{0021000}c_3^2c_4 + \mathbf{k}_{0020100}c_3^2c_5 + \mathbf{k}_{0020010}c_3^2c_6 + \mathbf{k}_{0020001}c_3^2c_7 + \mathbf{k}_{1002000}c_1c_4^2 + \\
&\mathbf{k}_{0102000}c_2c_4^2 + \mathbf{k}_{0012000}c_3c_4^2 + \mathbf{k}_{0002100}c_4^2c_5 + \mathbf{k}_{0002010}c_4^2c_6 + \mathbf{k}_{0002001}c_4^2c_7 + \mathbf{k}_{1000200}c_1c_5^2 + \\
&\mathbf{k}_{0100200}c_2c_5^2 + \mathbf{k}_{0010200}c_3c_5^2 + \mathbf{k}_{0001200}c_4c_5^2 + \mathbf{k}_{0000210}c_5^2c_6 + \mathbf{k}_{0000201}c_5^2c_7 + \mathbf{k}_{1000020}c_1c_6^2 + \\
&\mathbf{k}_{0100020}c_2c_6^2 + \mathbf{k}_{0010020}c_3c_6^2 + \mathbf{k}_{0001020}c_4c_6^2 + \mathbf{k}_{0000120}c_5c_6^2 + \mathbf{k}_{0000021}c_6^2c_7 + \mathbf{k}_{1000002}c_1c_7^2 + \\
&\mathbf{k}_{0100002}c_2c_7^2 + \mathbf{k}_{0010002}c_3c_7^2 + \mathbf{k}_{0001002}c_4c_7^2 + \mathbf{k}_{0000102}c_5c_7^2 + \mathbf{k}_{0000012}c_6c_7^2 + \mathbf{k}_{1110000}c_1c_2c_3 + \\
&\mathbf{k}_{1101000}c_1c_2c_4 + \mathbf{k}_{1100100}c_1c_2c_5 + \mathbf{k}_{1100010}c_1c_2c_6 + \mathbf{k}_{1100001}c_1c_2c_7 + \mathbf{k}_{0111000}c_2c_3c_4 + \\
&\mathbf{k}_{0110100}c_2c_3c_5 + \mathbf{k}_{0110010}c_2c_3c_6 + \mathbf{k}_{0110001}c_2c_3c_7 + \mathbf{k}_{0011100}c_3c_4c_5 + \mathbf{k}_{0011010}c_3c_4c_6 + \\
&\mathbf{k}_{0011001}c_3c_4c_7 + \mathbf{k}_{0001110}c_4c_5c_6 + \mathbf{k}_{0001101}c_4c_5c_7 + \mathbf{k}_{0000111}c_5c_6c_7 + \mathbf{k}_{1011000}c_1c_3c_4 + \\
&\mathbf{k}_{1001100}c_1c_4c_5 + \mathbf{k}_{1000110}c_1c_5c_6 + \mathbf{k}_{1000011}c_1c_6c_7 + \mathbf{k}_{0101100}c_2c_4c_5 + \mathbf{k}_{0100110}c_2c_5c_6 + \\
&\mathbf{k}_{0100011}c_2c_6c_7 + \mathbf{k}_{0010110}c_3c_5c_6 + \mathbf{k}_{0010011}c_3c_6c_7 + \mathbf{k}_{0001011}c_4c_6c_7 + \mathbf{k}_{1010100}c_1c_3c_5 + \\
&\mathbf{k}_{1010010}c_1c_3c_6 + \mathbf{k}_{1010001}c_1c_3c_7 + \mathbf{k}_{0101010}c_2c_4c_6 + \mathbf{k}_{0101001}c_2c_4c_7 + \mathbf{k}_{0010101}c_3c_5c_7 + \\
&\mathbf{k}_{1001010}c_1c_4c_6 + \mathbf{k}_{1001001}c_1c_4c_7 + \mathbf{k}_{1000101}c_1c_5c_7 + \mathbf{k}_{0100101}c_2c_5c_7. \qquad \text{(A5)}
\end{aligned}
$$

The equilibrium constants of three independent reactions were used to express the following equilibrium concentrations: $c_{1,\mathrm{eq}} = K_1^{-1}c_{2,\mathrm{eq}}c_{3,\mathrm{eq}}$, $c_{6,\mathrm{eq}} = \frac{K_2c_{2,\mathrm{eq}}c_{5,\mathrm{eq}}}{c_{4,\mathrm{eq}}}$, $c_{7,\mathrm{eq}} = K_3c_{4,\mathrm{eq}}$. They were introduced into the equilibrium form of the thermodynamic polynomial (A5) in which the rate is zero: $\mathbf{J}_{\mathrm{eq}} = \mathbf{0}$. Because the zero value should be valid at any equilibrium concentration, the following coefficients (which all are functions of temperature) are zero:

$\mathbf{k}_{0000000}, \mathbf{k}_{0100000}, \mathbf{k}_{0200000}, \mathbf{k}_{0300000}, \mathbf{k}_{0010000}, \mathbf{k}_{1200000}, \mathbf{k}_{0020000}, \mathbf{k}_{2100000}, \mathbf{k}_{0030000}, \mathbf{k}_{1020000}, \mathbf{k}_{2010000}, \mathbf{k}_{3000000}, \mathbf{k}_{0000100}, \mathbf{k}_{0000010}, \mathbf{k}_{0100010}, \mathbf{k}_{0010100}, \mathbf{k}_{0010010}, \mathbf{k}_{2000100}, \mathbf{k}_{2000010}, \mathbf{k}_{0200010}, \mathbf{k}_{0020100}, \mathbf{k}_{0020010}, \mathbf{k}_{1100010}, \mathbf{k}_{1010100}, \mathbf{k}_{1010010}, \mathbf{k}_{0000200}, \mathbf{k}_{0000020}, \mathbf{k}_{0000110}, \mathbf{k}_{1000200}, \mathbf{k}_{0010200}, \mathbf{k}_{1000020}, \mathbf{k}_{0100020}, \mathbf{k}_{0010020}, \mathbf{k}_{1000110}, \mathbf{k}_{0010110}, \mathbf{k}_{0000300}, \mathbf{k}_{0000030}, \mathbf{k}_{0000210}, \mathbf{k}_{0000120}$

and a number of others could be expressed, for example, as follows:

$\mathbf{k}_{0210000} = -\mathbf{k}_{1100000}K_1^{-1}$,

$\mathbf{k}_{0110000} = -\mathbf{k}_{1000000}K_1^{-1}$,

$\mathbf{k}_{0120000} = -\mathbf{k}_{1010000}K_1^{-1}$,

$\mathbf{k}_{1110000} = -\mathbf{k}_{2000000}K_1^{-1}$,

$\mathbf{k}_{0000001} = -\mathbf{k}_{0001000}K_3^{-1}$,

$\mathbf{k}_{0100001} = -\mathbf{k}_{0101000}K_3^{-1}$,

$\mathbf{k}_{0010001} = -\mathbf{k}_{0011000}K_3^{-1}$,

$\mathbf{k}_{2000001} = -\mathbf{k}_{2001000}K_3^{-1}$,

$\mathbf{k}_{0200001} = -\mathbf{k}_{0201000}K_3^{-1}$,

$\mathbf{k}_{0020001} = -\mathbf{k}_{0021000}K_3^{-1}$,

$\mathbf{k}_{1100001} = -\mathbf{k}_{1101000}K_3^{-1}$,

$\mathbf{k}_{1010001} = -\mathbf{k}_{1011000}K_3^{-1}$,

$\mathbf{k}_{0001001} = -\mathbf{k}_{0002000}K_3^{-1} - \mathbf{k}_{0000002}K_3$,

$\mathbf{k}_{1001001} = -\mathbf{k}_{1002000}K_3^{-1} - \mathbf{k}_{1000002}K_3$,

$\mathbf{k}_{0101001} = -\mathbf{k}_{0102000}K_3^{-1} - \mathbf{k}_{0100002}K_3$,

$\mathbf{k}_{0011001} = -\mathbf{k}_{0012000}K_3^{-1} - \mathbf{k}_{0010002}K_3,$
$\mathbf{k}_{0110010} = -\mathbf{k}_{1000010}K_1^{-1},$
$\mathbf{k}_{0001010} = -\mathbf{k}_{0100100}K_2^{-1} - \mathbf{k}_{0000011}K_3,$
$\mathbf{k}_{0000101} = -\mathbf{k}_{0001100}K_3^{-1},$
$\mathbf{k}_{0101010} = -\mathbf{k}_{0200100}K_2^{-1} - \mathbf{k}_{0100011}K_3,$
$\mathbf{k}_{0001101} = -\mathbf{k}_{0002100}K_3^{-1} - \mathbf{k}_{0000102}K_3,$
$\mathbf{k}_{0010101} = -\mathbf{k}_{0011100}K_3^{-1},$
$\mathbf{k}_{1000101} = -\mathbf{k}_{1001100}K_3^{-1},$
$\mathbf{k}_{0001110} = -\mathbf{k}_{0100200}K_2^{-1} - \mathbf{k}_{0000111}K_3,$
$\mathbf{k}_{0000201} = -\mathbf{k}_{0001200}K_3^{-1},$
$\mathbf{k}_{0001020} = -\mathbf{k}_{0100110}K_2^{-1} - \mathbf{k}_{0000021}K_3,$
$\mathbf{k}_{0110001} = -\mathbf{k}_{1001000}K_1^{-1}K_3^{-1} - \mathbf{k}_{1000001}K_1^{-1} - \mathbf{k}_{0111000}K_3^{-1},$
$\mathbf{k}_{0002001} = -\mathbf{k}_{0003000}K_3^{-1} - \mathbf{k}_{0000003}K_3^{2} - \mathbf{k}_{0001002}K_3,$
$\mathbf{k}_{0011010} = -\mathbf{k}_{1000100}K_1^{-1}K_2^{-1} - \mathbf{k}_{0110100}K_2^{-1} - \mathbf{k}_{0010011}K_3,$
$\mathbf{k}_{0002010} = -\mathbf{k}_{0000012}K_3^{2} - \mathbf{k}_{0101100}K_2^{-1} - \mathbf{k}_{0001011}K_3 - \mathbf{k}_{0100101}K_2^{-1}K_3,$
$\mathbf{k}_{1001010} = -\mathbf{k}_{1100100}K_2^{-1} - \mathbf{k}_{1000011}K_3.$

Introducing all these equalities into the original polynomial (A5) yields the final thermodynamic polynomial:

$$\mathbf{J} = \mathbf{k}_{0001000}(c_4 - K_3^{-1}c_7) + \mathbf{k}_{1000000}(c_1 - K_1^{-1}c_2c_3) + \mathbf{k}_{0101000}(c_2c_4 - K_3^{-1}c_2c_7) + \mathbf{k}_{0011000}(c_3c_4 - K_3^{-1}c_3c_7) + \mathbf{k}_{0100100}(c_2c_5 - K_2^{-1}c_4c_6) + \mathbf{k}_{0000011}(c_6c_7 - K_3c_4c_6) + \mathbf{k}_{0002000}(c_4^2 - K_3^{-1}c_4c_7) + \mathbf{k}_{0000002}(c_7^2 - K_3c_4c_7) + \mathbf{k}_{0001100}(c_4c_5 - K_3^{-1}c_5c_7) + \mathbf{k}_{2001000}(c_1^2c_4 - K_3^{-1}c_1^2c_7) + \mathbf{k}_{1100000}(c_1c_2 - K_1^{-1}c_2^2c_3) + \mathbf{k}_{0201000}(c_2^2c_4 - K_3^{-1}c_2^2c_7) + \mathbf{k}_{1010000}(c_1c_3 - K_1^{-1}c_2c_3^2) + \mathbf{k}_{0021000}(c_3^2c_4 - K_3^{-1}c_3^2c_7) + \mathbf{k}_{0000012}(c_6c_7^2 - K_3^2c_4^2c_6) + \mathbf{k}_{0101100}(c_2c_4c_5 - K_2^{-1}c_4^2c_6) + \mathbf{k}_{0001011}(c_4c_6c_7 - K_3c_4^2c_6) + \mathbf{k}_{0100101}(c_2c_5c_7 - K_2^{-1}K_3c_4^2c_6) + \mathbf{k}_{0003000}(c_4^3 - K_3^{-1}c_4^2c_7) + \mathbf{k}_{0000003}(c_7^3 - K_3^2c_4^2c_7) + \mathbf{k}_{0001002}(c_4c_7^2 - K_3c_4^2c_7) + \mathbf{k}_{0001200}(c_4c_5^2 - K_3^{-1}c_5^2c_7) + \mathbf{k}_{0100110}(c_2c_5c_6 - K_2^{-1}c_4c_6^2) + \mathbf{k}_{0000021}(c_6^2c_7 - K_3c_4c_6^2) + \mathbf{k}_{2000000}(c_1^2 - K_1^{-1}c_1c_2c_3) + \mathbf{k}_{1101000}(c_1c_2c_4 - K_3^{-1}c_1c_2c_7) + \mathbf{k}_{100010}(c_1c_6 - K_1^{-1}c_2c_3c_6) + \mathbf{k}_{1001000}(c_1c_4 - K_1^{-1}K_3^{-1}c_2c_3c_7) + \mathbf{k}_{1000001}(c_1c_7 - K_1^{-1}c_2c_3c_7) + \mathbf{k}_{0111000}(c_2c_3c_4 - K_3^{-1}c_2c_3c_7) + \mathbf{k}_{1000100}(c_1c_5 - K_1^{-1}K_2^{-1}c_3c_4c_6) + \mathbf{k}_{0110100}(c_2c_3c_5 - K_2^{-1}c_3c_4c_6) + \mathbf{k}_{0010011}(c_3c_6c_7 - K_3c_3c_4c_6) + \mathbf{k}_{0012000}(c_3c_4^2 - K_3^{-1}c_3c_4c_7) + \mathbf{k}_{0010002}(c_3c_7^2 - K_3c_3c_4c_7) + \mathbf{k}_{0100200}(c_2c_5^2 - K_2^{-1}c_4c_5c_6) + \mathbf{k}_{0000111}(c_5c_6c_7 - K_3c_4c_5c_6) + \mathbf{k}_{0002100}(c_4^2c_5 - K_3^{-1}c_4c_5c_7) + \mathbf{k}_{0000102}(c_5c_7^2 - K_3c_4c_5c_7) + \mathbf{k}_{1011000}(c_1c_3c_4 - K_3^{-1}c_1c_3c_7) + \mathbf{k}_{0200100}(c_2^2c_5 - K_2^{-1}c_2c_4c_6) + \mathbf{k}_{0100011}(c_2c_6c_7 - K_3c_2c_4c_6) + \mathbf{k}_{0102000}(c_2c_4^2 - K_3^{-1}c_2c_4c_7) + \mathbf{k}_{0100002}(c_2c_7^2 - K_3c_2c_4c_7) + \mathbf{k}_{0111000}(c_3c_4c_5 - K_3^{-1}c_3c_5c_7) + \mathbf{k}_{1100100}(c_1c_2c_5 - K_2^{-1}c_1c_4c_6) + \mathbf{k}_{1000011}(c_1c_6c_7 - K_3c_1c_4c_6) + \mathbf{k}_{1002000}(c_1c_4^2 - K_3^{-1}c_1c_4c_7) + \mathbf{k}_{1000002}(c_1c_7^2 - K_3c_1c_4c_7) + \mathbf{k}_{1001100}(c_1c_4c_5 - K_3^{-1}c_1c_5c_7). \quad \text{(A6)}$$

The individual terms can be interpreted as representing the mass-action rates of steps in the underlying reaction scheme, which is then:

Y = Z, **(RA1)**

AX = A + X, **(RA2)**

X + Y = X + Z, (RA3)

A + Y = A + Z, (RA4)

X + BY = Y + BX, **(RA5)**

$BX + Z = Y + B\,X,$ (RA6)

$2Y = Y + Z,$ (RA7)

$2Z = Y + Z,$ (RA8)

$Y + BY = BY + Z,$ (RA9)

$2AX + Y = 2AX + Z,$ (RA10)

$\mathbf{AX + X = 2X + A,}$ **(RA11)**

$2X + Y = 2X + Z,$ (RA12)

$AX + A = X + 2A,$ (RA13)

$2A + Y = 2A + Z,$ (RA14)

$BX + 2Z = 2Y + BX,$ (RA15)

$\mathbf{X + Y + BY = 2Y + BX,}$ **(RA16)**

$Y + BX + Z = 2Y + BX,$ (RA17)

$X + BY + Z = 2Y + BX,$ (RA18)

$3Y = 2Y + Z,$ (RA19)

$3Z = 2Y + Z,$ (RA20)

$Y + 2Z = 2Y + Z,$ (RA21)

$Y + 2BY = 2BY + Z,$ (RA22)

$X + BY + BX = Y + 2BX,$ (RA23)

$2BX + Z = Y + 2BX,$ (RA24)

$2AX = AX + X + A,$ (RA25)

$AX + X + Y = AX + X + Z,$ (RA26)

$AX + BX = X + A + BX,$ (RA27)

$AX + Y = X + A + Z,$ (RA28)

$AX + Z = X + A + Z,$ (RA29)

$X + A + Y = X + A + Z,$ (RA30)

$AX + BY = A + Y + BX,$ (RA31)

$X + A + BY = A + Y + BX,$ (RA32)

$A + BX + Z = A + Y + BX,$ (RA33)

$A + 2Y = A + Y + Z,$ (RA34)

$A + 2Z = A + Y + Z,$ (RA35)

$X + 2BY = Y + BY + BX,$ (RA36)

$BY + BX + Z = Y + BY + BX,$ (RA37)

$2Y + BY = Y + BY + Z,$ (RA38)

$BY + 2Z = Y + BY + Z,$ (RA39)

$AX + A + Y = AX + A + Z,$ (RA40)

$2X + BY = X + Y + BX,$ (RA41)

$X + BX + Z = X + Y + BX,$ (RA42)

$X + 2Y = X + Y + Z,$ (RA43)

$X + 2Z = X + Y + Z,$ (RA44)

$A + Y + BY = A + BY + Z,$ (RA45)

$AX + X + BY = AX + Y + BX,$ (RA46)

AX + BX + Z = AX + Y + BX, (RA47)

AX + 2Y = AX + Y + Z, (RA48)

AX + 2Z = AX + Y + Z, (RA49)

AX + Y + BY = AX + BY + Z. (RA50)

The steps in bold belong either to the selected independent reactions or to Lotka's scheme (R4) in the main text. If the rate coefficients of all other steps are set equal to zero, a simplified reaction scheme follows with a rate equation in the form of the thermodynamic polynomial given by eq. (22) in the main text where the indexing of rate coefficients was simplified as follows:

$\mathbf{k}_1 = \mathbf{k}_{2000000}, \mathbf{k}_2 = \mathbf{k}_{0100100}, \mathbf{k}_3 = \mathbf{k}_{0001000}, \mathbf{k}_4 = \mathbf{k}_{1100000}, \mathbf{k}_5 = \mathbf{k}_{0101100}$

The chemical affinities in this simplified scheme are obtained from their general definition [9] (p. 181) as:

$$A^1 = -\mu_{\mathrm{AX}} + \mu_{\mathrm{X}} + \mu_{\mathrm{A}},\ A^2 = -\mu_{\mathrm{X}} + \mu_{\mathrm{Y}} - \mu_{\mathrm{BY}} + \mu_{\mathrm{BX}},\ A^3 = -\mu_{\mathrm{Y}} + \mu_{\mathrm{Z}}. \quad \text{(A7)}$$

The basis vectors necessary to express the constitutional affinities [9] (p. 152) are:

$$f_1 = (1,0,1,0,0,0,0),\ f_2 = (1,1,0,0,0,1,0),\ f_3 = (0,0,0,1,1,0,1),\ f_4 = (0,0,0,0,1,1,0). \quad \text{(A8)}$$

The contravariant components $f^{\sigma\tau}$ of the metric tensor [9] (p. 295) obtained by inverting the metric tensor in covariant components ($f_{\sigma\tau} = \mathbf{f}_\sigma.\mathbf{f}_\tau$), are also necessary to express the constitutional constitutive affinities. In matrix form, these contravariant components are:

$$\begin{bmatrix} 12/19 & -5/19 & -1/19 & 3/4 \\ -5/19 & 10/19 & 2/19 & -6/4 \\ -1/19 & 2/19 & 8/19 & -5/4 \\ 3/19 & -6/19 & -5/19 & 15/4 \end{bmatrix}. \quad \text{(A9)}$$

The constitutional affinities then are:

$$B^1 = \frac{7}{19}\mu_{\mathrm{AX}} - \frac{5}{19}\mu_{\mathrm{X}} + \frac{12}{19}\mu_{\mathrm{A}} - \frac{1}{19}\mu_{\mathrm{Y}} + \frac{53}{76}\mu_{\mathrm{BY}} + \frac{37}{76}\mu_{\mathrm{BX}} - \frac{1}{19}\mu_{\mathrm{Z}},$$
$$B^2 = \frac{5}{19}\mu_{\mathrm{AX}} + \frac{10}{19}\mu_{\mathrm{X}} - \frac{5}{19}\mu_{\mathrm{A}} + \frac{2}{19}\mu_{\mathrm{Y}} - \frac{106}{76}\mu_{\mathrm{BY}} - \frac{74}{76}\mu_{\mathrm{BX}} + \frac{2}{19}\mu_{\mathrm{Z}},$$
$$B^3 = \frac{1}{19}\mu_{\mathrm{AX}} + \frac{2}{19}\mu_{\mathrm{X}} - \frac{1}{19}\mu_{\mathrm{A}} + \frac{8}{19}\mu_{\mathrm{Y}} - \frac{63}{76}\mu_{\mathrm{BY}} - \frac{87}{76}\mu_{\mathrm{BX}} + \frac{8}{19}\mu_{\mathrm{Z}},$$
$$B^4 = -\frac{3}{19}\mu_{\mathrm{AX}} - \frac{6}{19}\mu_{\mathrm{X}} + \frac{3}{19}\mu_{\mathrm{A}} - \frac{5}{19}\mu_{\mathrm{Y}} + \frac{265}{76}\mu_{\mathrm{BY}} + \frac{261}{76}\mu_{\mathrm{BX}} - \frac{5}{19}\mu_{\mathrm{Z}}. \quad \text{(A10)}$$

From eqs. (A8) and (A10) the decomposition of the chemical potentials into affinities [9] (p. 181) is obtained:

$$\mu_{\mathrm{AX}} = -\frac{7}{19}A^1 - \frac{2}{19}A^2 - \frac{1}{19}A^3 + B^1 + \frac{406}{361}B^2 + \frac{45}{361}B^3 + \frac{90}{361}B^4,$$
$$\mu_{\mathrm{X}} = \frac{5}{19}A^1 - \frac{4}{19}A^2 - \frac{2}{19}A^3 + \frac{451}{361}B^2 + \frac{90}{361}B^3 + \frac{180}{361}B^4,$$
$$\mu_{\mathrm{A}} = \frac{7}{19}A^1 + \frac{2}{19}A^2 + \frac{1}{19}A^3 + B^1 - \frac{45}{361}B^2 - \frac{45}{361}B^3 - \frac{90}{361}B^4,$$
$$\mu_{\mathrm{Y}} = \frac{1}{19}A^1 + \frac{3}{19}A^2 - \frac{8}{19}A^3 + \frac{75}{361}B^2 + \frac{436}{361}B^3 + \frac{150}{361}B^4,$$
$$\mu_{\mathrm{BY}} = -\frac{2}{19}A^1 - \frac{6}{19}A^2 - \frac{3}{19}A^3 - \frac{150}{361}B^2 + \frac{211}{361}B^3 + \frac{61}{361}B^4,$$
$$\mu_{\mathrm{BX}} = \frac{2}{19}A^1 + \frac{6}{19}A^2 + \frac{3}{19}A^3 + \frac{226}{361}B^2 - \frac{135}{361}B^3 + \frac{91}{361}B^4,$$
$$\mu_{\mathrm{Z}} = \frac{1}{19}A^1 + \frac{3}{19}A^2 + \frac{11}{19}A^3 + \frac{75}{361}B^2 + \frac{436}{361}B^3 + \frac{150}{361}B^4. \quad \text{(A11)}$$

The relationships in (A11) are introduced into eq. (23) in the main text. Then, the transformation of the reaction rate into the function of affinities follows finally and looks like this:

$$\mathbf{J} = -\mathbf{k}_1 \exp\frac{-\mu_{\mathrm{AX}}^{\mathrm{o}}}{RT}\exp\frac{\mu_{\mathrm{AX}}}{RT}\left(\exp\frac{A^1}{RT}-1\right) - \mathbf{k}_2 \exp\frac{-\mu_{\mathrm{X}}^{\mathrm{o}}-\mu_{\mathrm{BY}}^{\mathrm{o}}}{RT}\exp\frac{C_1}{RT}\left(\exp\frac{A^2}{RT}-1\right) -$$
$$\mathbf{k}_3 \exp\frac{-\mu_{\mathrm{Y}}^{\mathrm{o}}}{RT}\exp\frac{\mu_{\mathrm{Y}}}{RT}\left(\exp\frac{A^3}{RT}-1\right) - \mathbf{k}_4 \exp\frac{-\mu_{\mathrm{AX}}^{\mathrm{o}}-\mu_{\mathrm{X}}^{\mathrm{o}}}{RT}\exp\frac{C_2}{RT}\left(\exp\frac{A^1}{RT}-1\right) -$$
$$\mathbf{k}_5 \exp\frac{-\mu_{\mathrm{X}}^{\mathrm{o}}-\mu_{\mathrm{BY}}^{\mathrm{o}}-\mu_{\mathrm{Y}}^{\mathrm{o}}}{RT}\exp\frac{C_3}{RT}\left(\exp\frac{A^2}{RT}-1\right) \quad \text{(A12)}$$

where

$$C_1 = \frac{3}{19}A^1 - \frac{10}{19}A^2 - \frac{5}{19}A^3 + \frac{301}{361}B^2 + \frac{301}{361}B^3 + \frac{241}{361}B^4,$$
$$C_2 = -\frac{2}{19}A^1 - \frac{6}{19}A^2 - \frac{3}{19}A^3 + B^1 + \frac{857}{361}B^2 + \frac{135}{361}B^3 + \frac{270}{361}B^4,$$
$$C_3 = \frac{4}{19}A^1 - \frac{7}{19}A^2 - \frac{13}{19}A^3 + \frac{376}{361}B^2 + \frac{737}{361}B^3 + \frac{391}{361}B^4.$$

and the corresponding relationships from (A11) should be substituted instead of the chemical potentials remaining in (A12). Note, that in equilibrium, where $A^i = 0$, the rate is zero, as it should be.

The matrix of the quadratic form in the main text (24), following from (A12) is:

$$\begin{bmatrix} \frac{\partial J_1}{\partial A^1} & (\frac{\partial J_1}{\partial A^2}+\frac{\partial J_2}{\partial A^1})/2 & (\frac{\partial J_1}{\partial A^3}+\frac{\partial J_3}{\partial A^1})/2 \\ & \frac{\partial J_2}{\partial A^2} & (\frac{\partial J_2}{\partial A^3}+\frac{\partial J_3}{\partial A^2})/2 \\ & & \frac{\partial J_3}{\partial A^3} \end{bmatrix}_{\mathrm{eq}}$$

(the matrix is symmetric; therefore only its upper diagonal form is shown).

References (from the main text)

9. Pekař, M. Thermodynamic framework for design of reaction rate equations and schemes. *Collect. Czech. Chem. Commun.* **2009**, *74*, 1375–1401.
10. Pekař, M.; Samohýl, I. *The Thermodynamics of Linear Fluids and Fluid Mixtures*. Springer: Cham, Switzerland, 2014.
16. Samohýl, I.; Malijevský, A. Phenomenological derivation of mass-action law of homogeneous chemical kinetics. *Collect. Czech. Chem. Commun.* **1976**, *41*, 2131–2142.
